# Long-term follow-up of a patient with Parkinson’s disease under nursing care after replacement of fixed implant-supported prostheses with an implant overdenture: a case report

**DOI:** 10.1186/s40729-024-00557-8

**Published:** 2024-07-29

**Authors:** Kana Tokumoto, Takuya Mino, Ikue Tosa, Ko Omori, Michiyo Yamamoto, Kazuki Takaoka, Kenji Maekawa, Takuo Kuboki, Hiromitsu Kishimoto

**Affiliations:** 1 Department of Oral and Maxillofacial Surgery, School of Medicine, Hyogo Medical University, Nishinomiya, Japan; 2 Okayama University Dental School, Okayama, Japan; 3 Department of Removable Prosthodontics and Occlusion, Osaka Dental University, 1-5-17 Otemae, Chuo-Ku, Osaka, 540-0008 Japan; 4 Department of Oral Rehabilitation and Regenerative Medicine, Dentistry and Pharmaceutical Sciences, Okayama University Graduate School of Medicine, Okayama, Japan; 5 Dental Clinic, AINOSATO Clinic, Okayama, Japan; 6 Department of Oral and Maxillofacial Surgery, Shiga University of Medical Science, Shiga, Japan

**Keywords:** Parkinson’s disease, Older people, Implant overdenture, Nursing homes, Implant-related troubles, Peri-implantitis

## Abstract

**Background:**

In older patients with progressive neurodegeneration, replacing fixed implant-supported prostheses (FIP) with implant overdentures (IOD) has been proposed to prevent future mucosal injury and create an oral environment that is easier for caregivers to clean. However, there have been no reports on the progress after replacing FIP with IOD. In this report, we present the progress of an older patient with Parkinson’s disease in whom FIP was replaced with IOD.

**Case presentation:**

An 81-year-old male patient with Parkinson’s disease presented to our outpatient clinic with bruxism and crossbites. FIPs, with five Brånemark system implants, were placed in the bilateral lower molars. The FIP was replaced with an IOD with two locator attachments to create an oral environment that was easier for caregivers to clean and allow easy recovery of masticatory function if residual teeth were fractured in the care environment. As his systemic condition deteriorated, treatment was changed from outpatient to in-home visits. During dental care visits, professional oral cleaning and denture repair were continued, and good nutritional status was maintained. However, the patient developed cholecystitis and was hospitalized. During hospitalization, gastrostomy was performed because he developed aspiration pneumonia. After discharge from the hospital, the patient remained in bed all day and could not wear an IOD, resulting in buccal mucosa ulceration due to abrasion of the locator abutment. We decided to replace the abutment with cover screws; however, not all the implants could sleep submucosally. Although regular oral cleaning was resumed, new ulcers developed even when cover screws were installed. Additionally, swelling and drainage were observed at the peri-implant mucosal site where peri-implantitis had once occurred during an outpatient visit. The patient was readmitted to the hospital for a urinary tract infection, and subsequent visits were abandoned.

**Conclusions:**

By replacing FIP with IOD in an older patient with Parkinson’s disease, we addressed a barrier to caregiver-provided oral management. The removable prosthesis facilitated smooth oral care by caregivers and functional recovery in the event of trouble with residual teeth. However, it could not completely avoid the recurrence of buccal mucosal ulcers or peri-implantitis.

## Background

In late-stage older patients with neurodegenerative diseases, such as dementia and Parkinson’s disease, intraoral remaining teeth or fixed prostheses may be damaging the buccal or alveolar mucosa [1, 2]. This problem can be addressed by extracting the causative teeth and remaining only the tooth root without coronal structure. However, many patients are unable to undergo these treatments because of severe systemic conditions for tooth extraction, high risk of aspiration during cutting off the coronal structure of tooth, and an insufficient understanding of the need for treatment. Attempts have also been made to wear oral appliances to prevent mucosal damage; however, impressions made with conventional materials could pose a risk of aspiration or choking. Therefore, we attempted to address these issues.

Previous studies have reported that fixed implant prostheses exhibit higher mastication ability [3], better nutritional status [4], and higher levels of oral health-related quality of life [5] than do conventional removable dentures. Thus, implant prostheses are considered to have a high potential for maintaining nutritional intake in older people. Owing to the excellent treatment outcomes and high survival rate of oral implants, the number of implant prostheses remaining in the oral cavity of older people is increasing. However, negative opinions exist regarding the placement of fixed implant superstructures up to the late older stage because they are difficult for caregivers to clean and can potentially damage the oral mucosa [6–8].

Therefore, to receive the benefits of such implant treatments and simultaneously avoid the risk of mucosal damage, it has been proposed that fixed implant prostheses be replaced with implant overdentures (IOD) before entering the terminal life stage [9]. The replacement of fixed implant prostheses with IODs not only avoids the risk of mucosal damage but also facilitates oral cleaning by caregivers and provides support and maintenance functions for attachments connected to the implant. Therefore, masticatory function is expected to improve compared with that with conventional removable dentures [4]. However, to date, no clinical reports have included systemic changes associated with a progressive level of nursing care after replacement with IOD. Establishing treatment guidelines for patients with implants requiring nursing care will be possible upon clarification of whether replacement with IODs maintains masticatory ability, helps in oral care and management by caregivers, and mitigates issues such as mucosal damage [10].

This clinical report presents the progress of a patient with Parkinson’s disease who underwent care through the replacement of a fixed implant prosthesis with an IOD to enable oral management in preparation for disease progression. The need for ethics approval was waived for this case report.

## Case presentation

### Patient

An 81-year-old man visited our hospital in 2016, complaining of difficulty in singing. He was a professional vocalist, and singing was his purpose. His medical history included hypertension since 2003 and diabetes mellitus since 2003, both of which were controlled with oral medications. His hemoglobin A1c level was 6.2% (The National Glycohemoglobin Standardization Program) at the time of initial visit. He was diagnosed with depression in 2009, Parkinson’s disease in 2011, and dementia in 2014. His Hoehn and Yahr Scale score was stage III. Bradykinesia and rigidity were also observed. He had already started treatment with L-dopa and a monoamine oxidase B inhibitor. His body mass index was 28.0 (kg/m^2^) at that time. He required nursing care of support level 2 according to the Long-Term Care Insurance Act in Japan [11] and received daytime care.

**Fig. 1:**
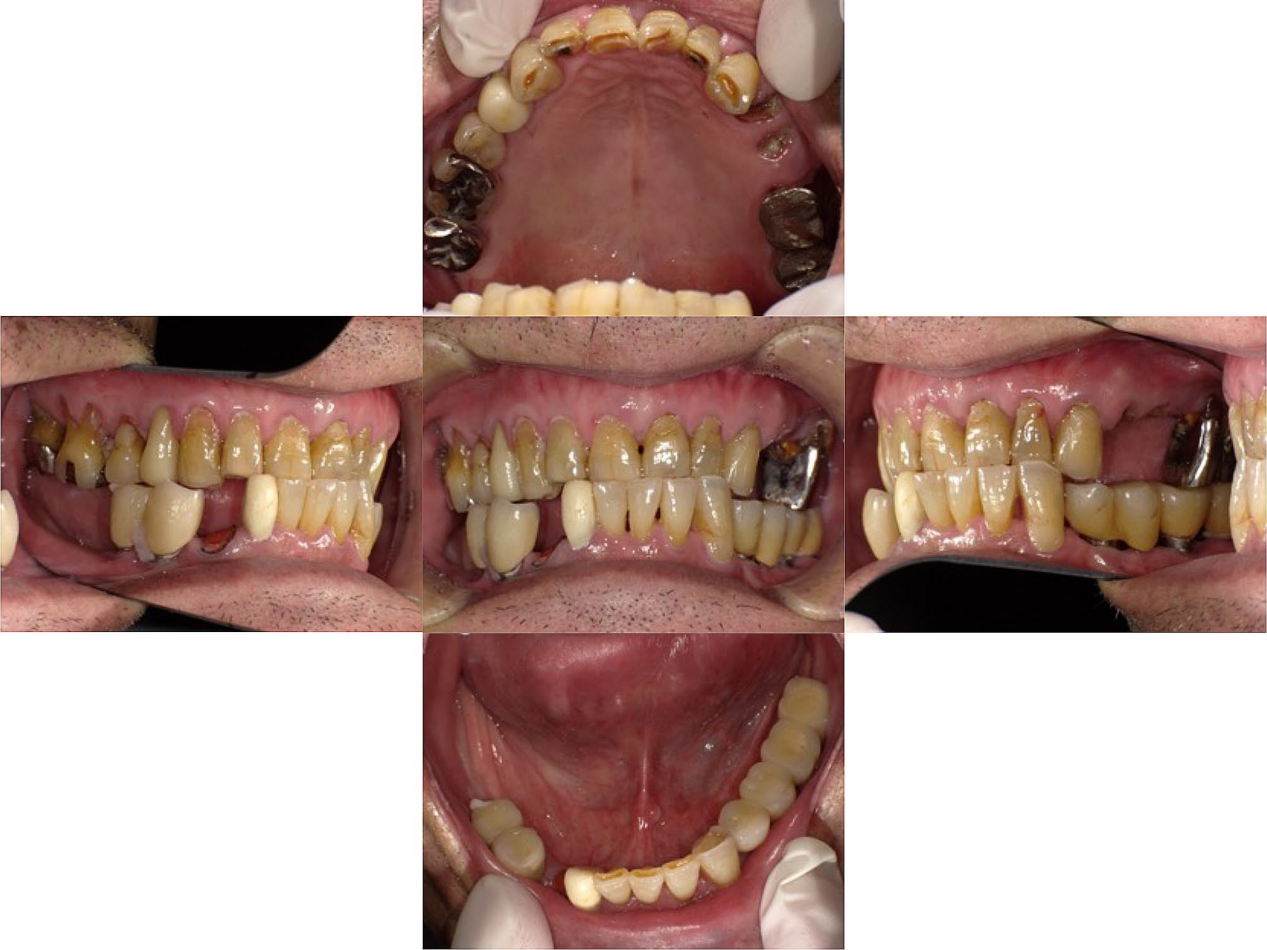
An intraoral photograph obtained at the patient’s first visit

**Fig. 2:**
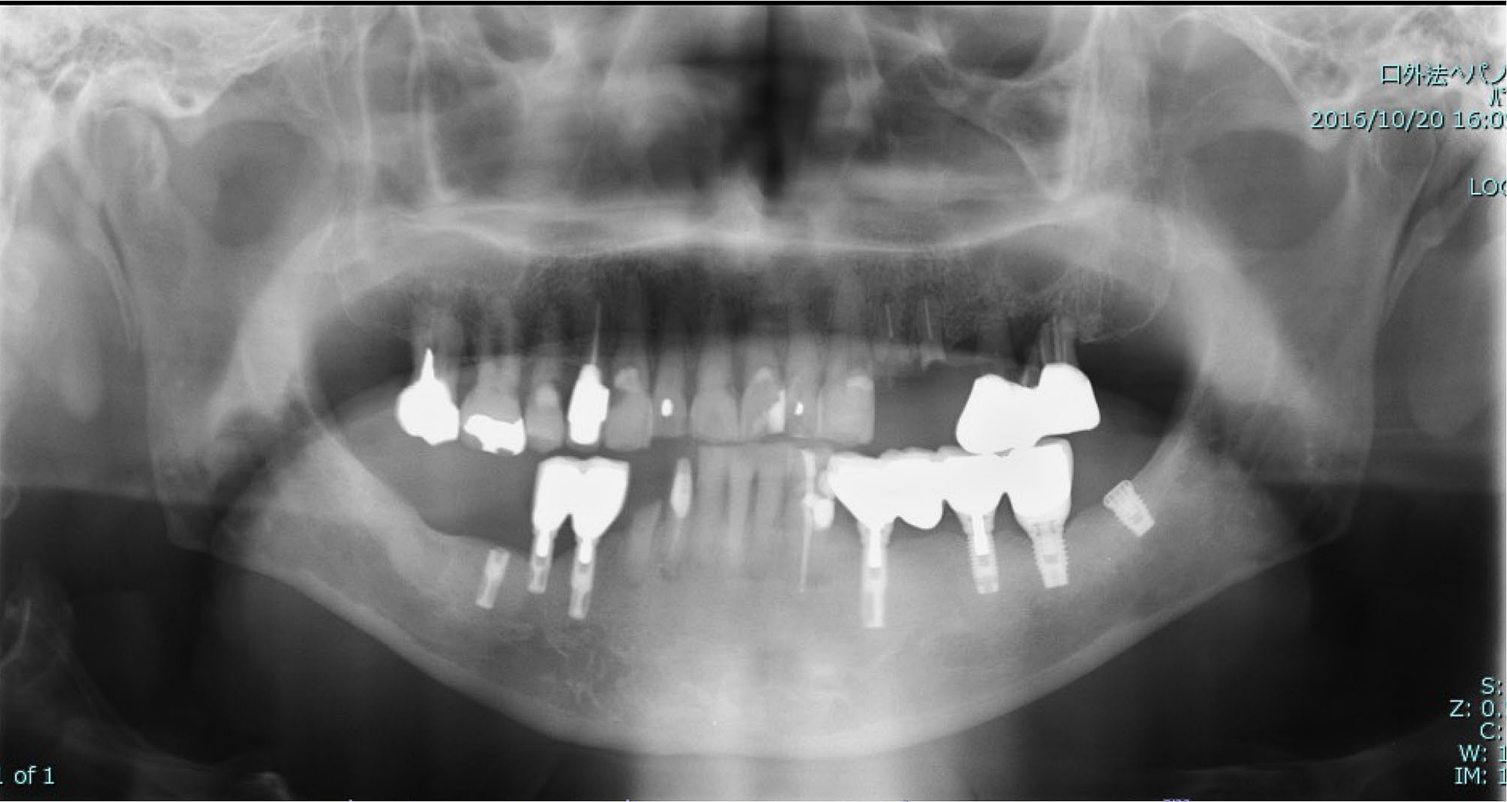
A panoramic radiograph image obtained at the patient’s first visit

Intra-oral examination revealed that #24, #25, and #43 had residual root status, and root surface caries were observed at #11, #13, #15, #16, #21, #22, and #23. The intermaxillary relationship was crossbite; furthermore, tooth wear was observed in almost all teeth (Fig. 1). Panoramic radiographs showed periapical lesions in #24, #25, #43, and #44, and severe bone resorption in #26 and #27 (Fig. 2). The patient had undergone implant treatment approximately 20 years prior (#34: Brånemark MkII 3.3 × 13 mm, #36: Brånemark MkII 3.75 × 10 mm, #37: Brånemark Mk IV 5 × 7 mm, #38: Brånemark MK II 5 × 7 mm, #44: Brånemark MkII 3.75 × 13 mm, #45: Brånemark MkII 3.75 × 10 mm, #46: Brånemark MkII 3.75 × 13 mm, Nobel Biocare). Fixed implant-supported superstructures were installed in #44–#45 and #34–#36–#37 regions. However, fixture #46 fractured in 2011. Additionally, implant fixtures #46 and #38 were asleep submucosally (Fig. 2).

### Patient problems and treatment plan

The patient’s oral problems included (1) tooth fractures at #24, #25, and #43; (2) chronic marginal periodontitis at #26 and #27; (3) apical periodontitis at #24, #25, and #42; and (4) root surface caries at #11, #13, #15, #16, #21, #22, and #23. Additionally, the patient’s crossbite and bruxism habits may have been related to a history of tooth fractures. Problems related to the patient’s systemic condition included dysfunction of the perioral muscles due to Parkinson’s disease and cognitive impairment due to dementia.

The following prosthetic treatment plans for the regions #24–#27 and #42–#43 were proposed to the patient: (1) application of implant-supported fixed prostheses with additional implant placement, (2) application of a conventional removable partial denture for the maxilla and an implant-supported fixed partial denture without additional implant placement (#42 cantilever), and (3) application of a conventional removable partial denture for the maxilla and IOD for the mandible after removing the implant superstructures #34–#37 and #44–#45.

A discussion was conducted with the patient and his wife, and they preferred to use a conventional removable partial denture for the maxilla and an IOD for the mandible. Informed consent has been obtained from the patient and his representative to publish the treatment process as a case report.

## Treatment process

**Fig. 3:**
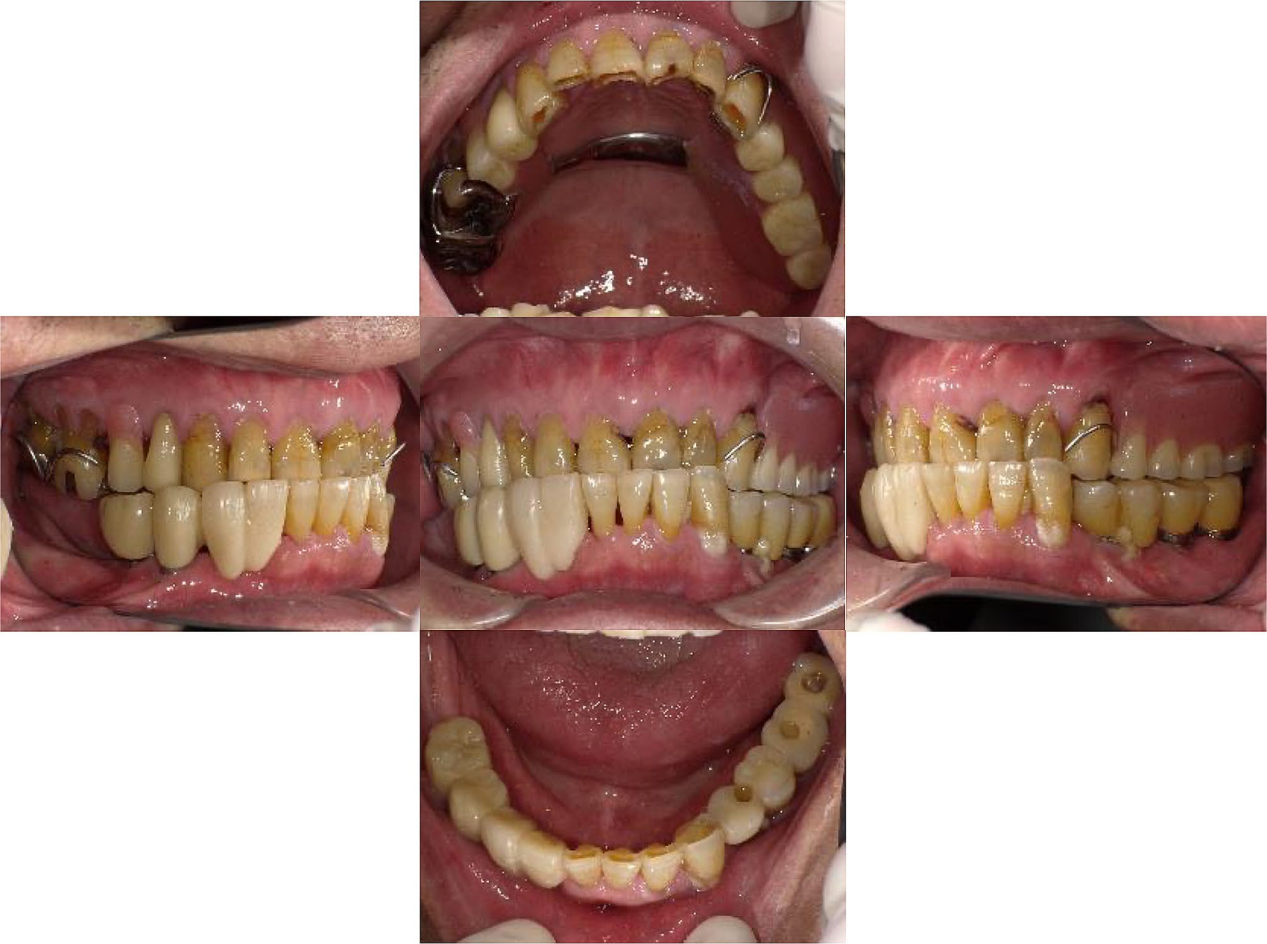
An intraoral photograph obtained after the implant-supported superstructures were transferred from cement-retained to screw-retained

**Fig. 4:**
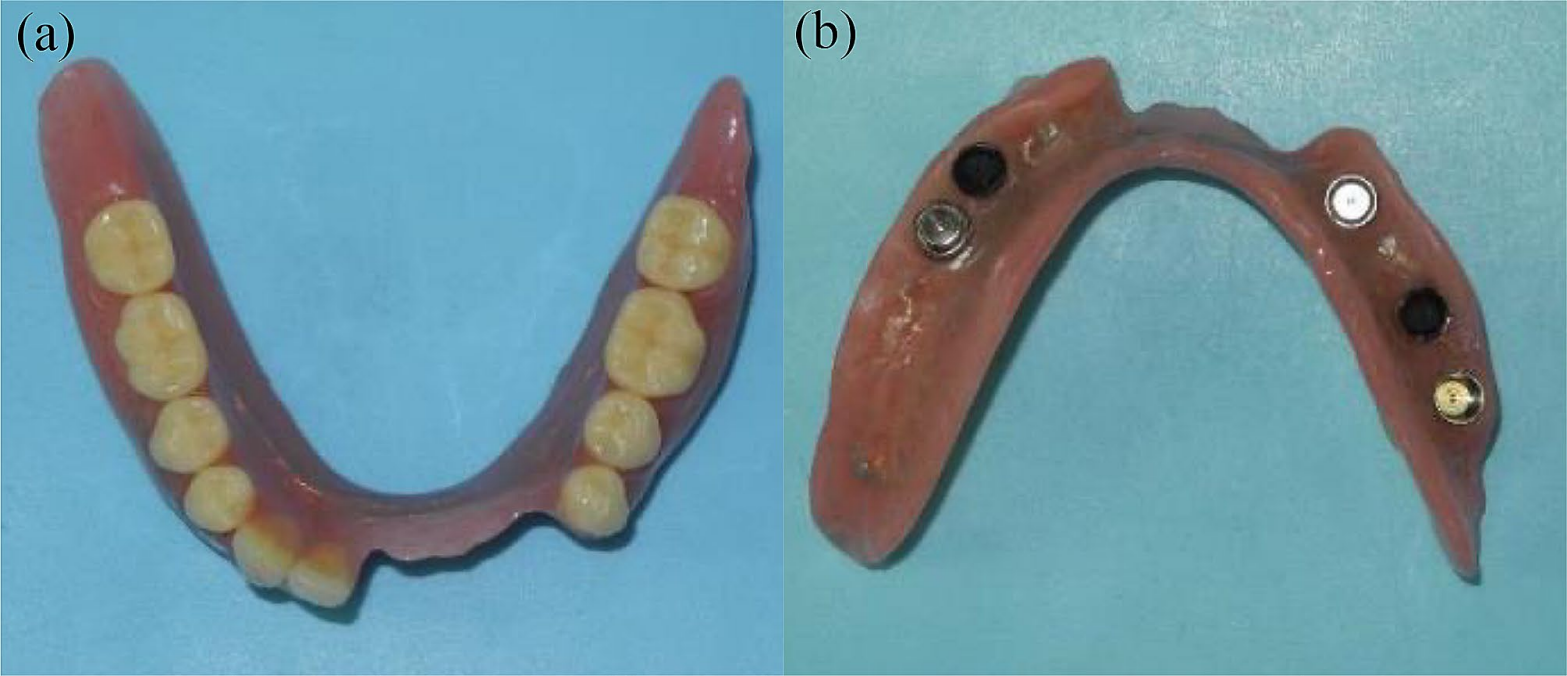
Fabricated implant overdenture for the mandible. (**a**) Polished side. (**b**) Basal side. Retention disks of 0.5 kg for locator attachments were installed at #36 and #44

Teeth #24, #25, #26, #42, and #43 were extracted. Maxillary removable partial dentures were installed in regions #15, #24, and #27. #15 fractured during the denture fabrication process and was preserved under the denture base in the residual root condition. As the patient was eager to improve his singing difficulties, a palatal bar was applied as a major connector to reduce discomfort during speech. In the mandible, the #44–#45 superstructure was removed, and a provisional fixed implant-supported restoration (#42–#43 cantilever) was installed to temporarily restore masticatory function. After the replacement of the cement-retained superstructure of #34–#37 with screw-retained superstructures (Fig. 3), the IOD fabrication was started. The IOD for regions #34–37 and #42–47 were successfully installed in September 2017 (Fig. 4). Locator attachments (Locator^®^, Nobel Biocare) were applied as implant-support attachments and Locator^®^ retention disks (Nobel Biocare) were installed at #36 and #44. After installation, the patient expressed satisfaction with singing and eating.

## Follow-up

### At the outpatient clinic

After removable dentures for the maxilla and IOD for the mandible were installed, regular follow-up visits were continued every month. During the follow-up period, professional oral care was provided, and the dentures were repaired when the remaining teeth were fractured by adding artificial teeth. Since January 2018, the patient has required a wheelchair to go out, and his nursing care level was changed from support level 2 to long-term care level 1. As the patient gradually became unable to wear the dentures, we provided repeated instructions on installing and withdrawing them.

In January 2019, wearing-off phenomenon which means that the Parkinson’s drugs are no longer effective as they used to be occurred as the patient’s general condition deteriorated. His nursing care level changed from long-term care level 1 to level 3. At that time, the locator abutment on #36 loosened, and the retention of the IOD decreased. Therefore, the retention disk of #36 was removed, and new retention disks (0 kg) were attached to #34 and #44. However, the retention disk of #44 was changed from 0 kg to 0.5 kg because the retention force was weak and easily detached. Additionally, we offered the patient’s wife help with wearing and removing the IOD and performing oral cleaning at home. In October 2019, drainage from the peri-implant sulcus of #45 was observed; however, the patient did not complain of pain. Based on the diagnosis of peri-implantitis, #45 was cleaned, and local antibiotic therapy was administered.

## Home-visit dental care and hospitalization

**Fig. 5:**
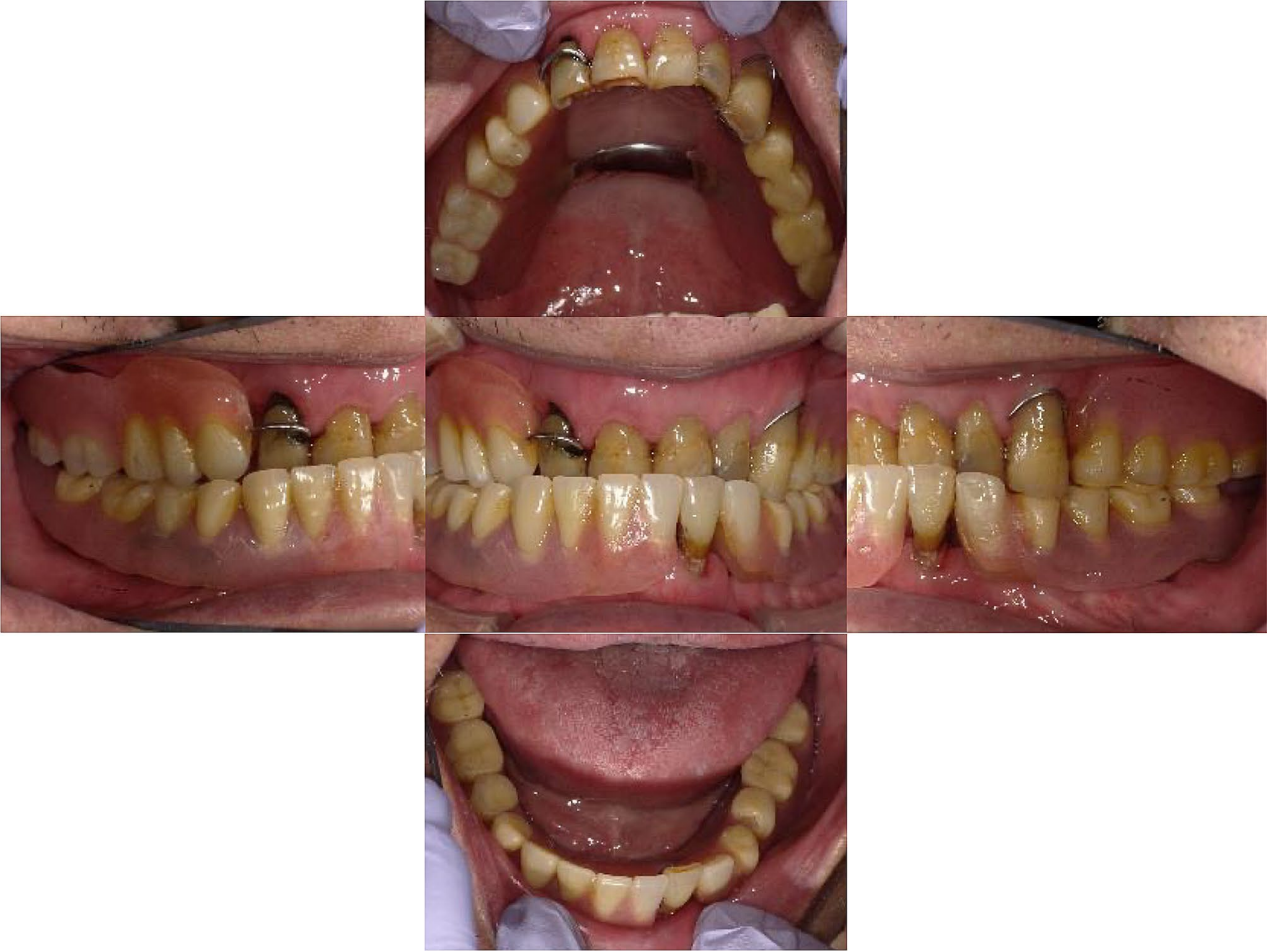
An intraoral photograph taken at the last visit to the outpatient clinic

**Fig. 6:**
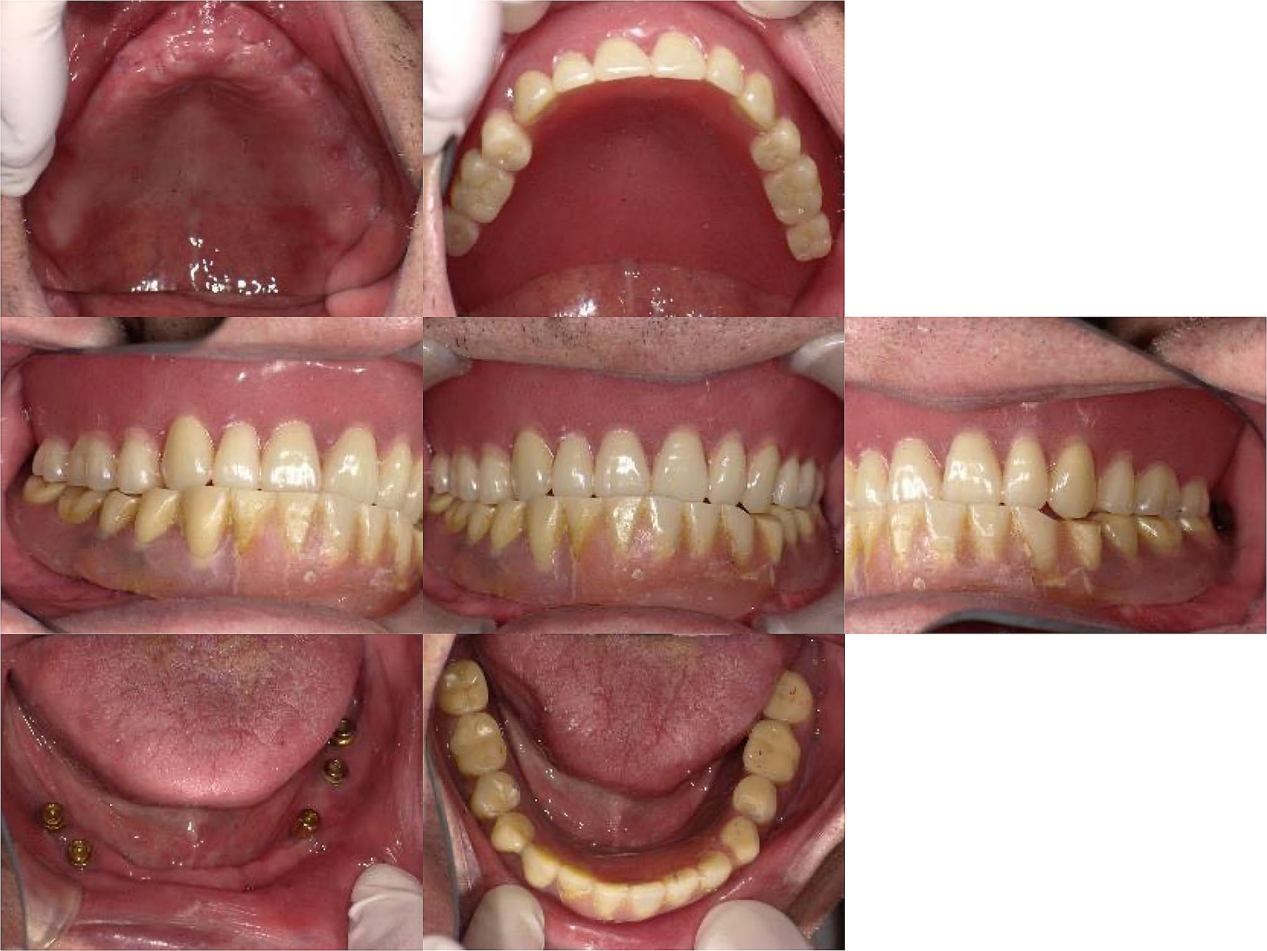
An intraoral photograph taken before hospitalization owing to cholecystitis

Considering the patient’s general condition, we decided to transition to home-visit dental care in 2020 (Fig. 5). Professional oral care was provided twice a month in January 2020; however, only teeth #11, #12, #21, #22, and #32 remained. Almost all fractures were caused by severe root caries, and the patient was predicted to become completely edentulous in the near future. Therefore, the decision was made to fabricate a new maxillary denture with a resin base covering the palatal area; however, immediately after fabrication of the denture, draining from the residual tooth area began. Subsequently, all teeth were extracted, and a new complete denture for the maxilla was delivered (Fig. 6). After the delivery of the new complete denture, the patient encountered no difficulty eating or enjoying singing. In January 2021, the patient’s nursing care level had deteriorated to a long-term care level 4. He remained sitting on a chair almost all day but could walk while using the handrail at home. His body mass index was 27.9 (kg/m^2^), which did not change significantly from the first visit. Although the patient’s wife was given oral cleaning instructions, oral cleaning for him was not easy for her because she was not a professional caregiver.

The patient was hospitalized for acute cholecystitis in May 2021 but was unable to continue home-visit dental care because of the restrictions on in-hospital visits due to the coronavirus disease pandemic. During hospitalization, the patient developed aspiration pneumonia and underwent gastrostomy.

## Resumption of home-visit dental treatment

**Fig. 7:**
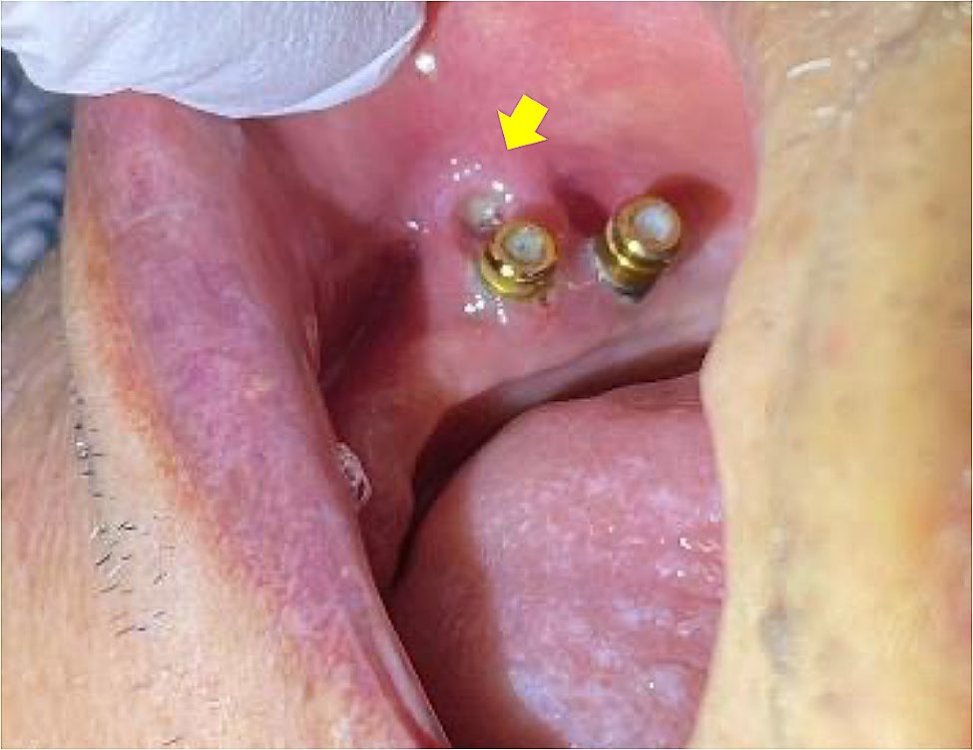
An intraoral photograph taken at the time of resuming home-visit dental treatment. An ulcer (yellow arrow) has developed at the right buccal mucosa due to abrasion of the locator abutment of #44

**Fig. 8:**
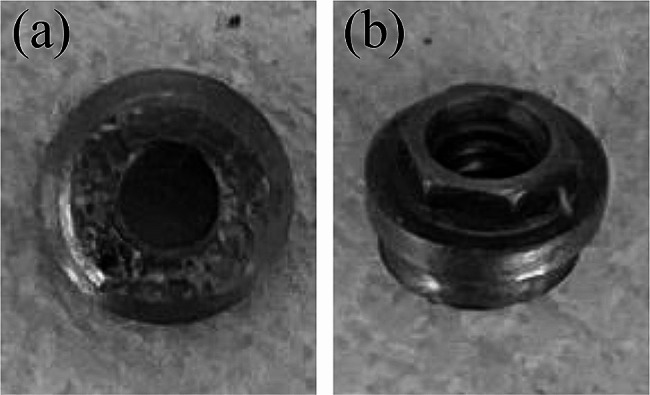
Fractured neck part of the fixture at #37. (**a**) Cut-off side. (**b**) Platform side

**Fig. 9:**
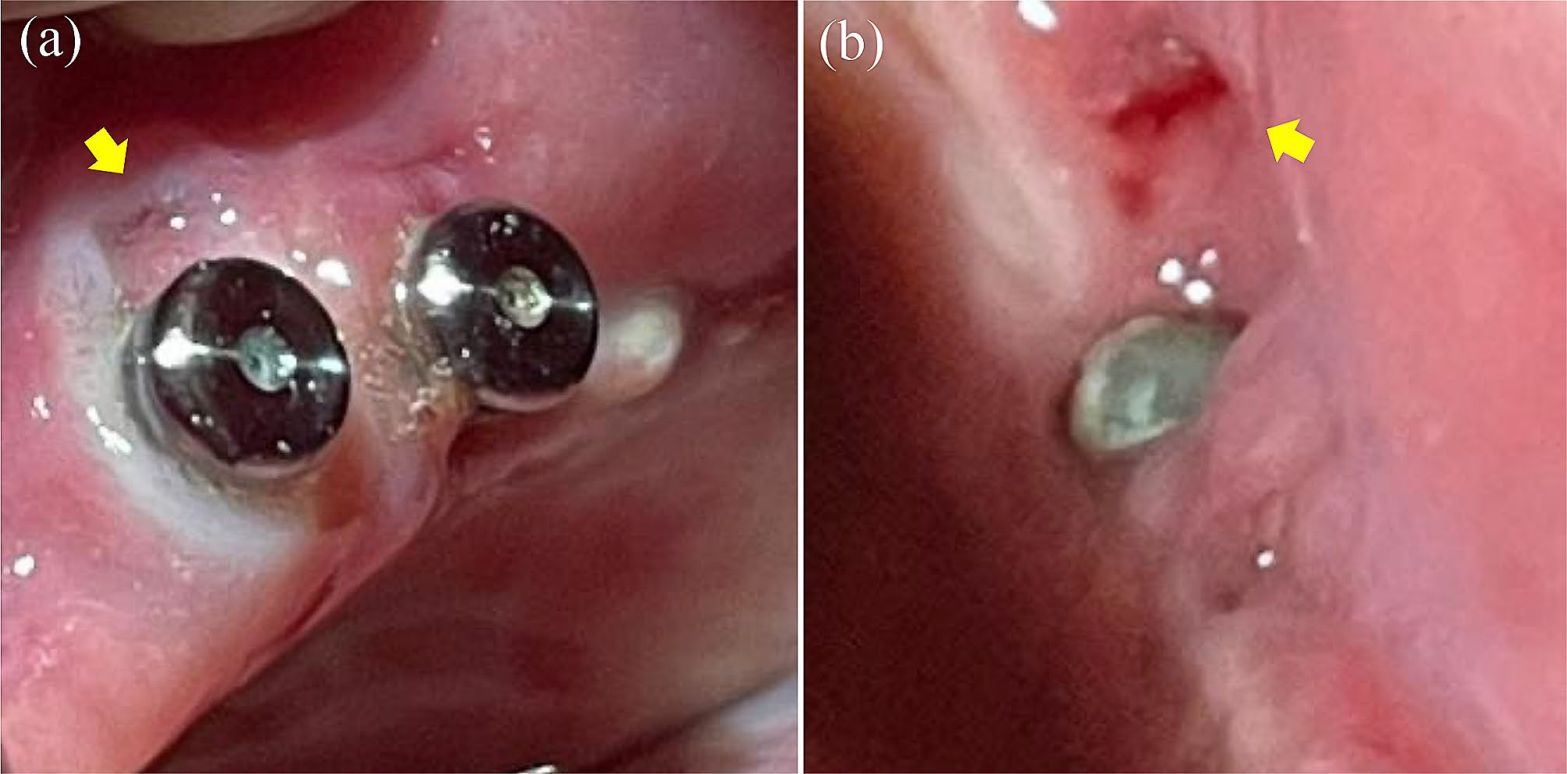
Ulcers that have re-developed around implants. (**a**) An ulcer (yellow arrow) that re-developed on the buccal mucosa beside #44. (**b**) An ulcer (yellow arrow) that re-developed on the buccal mucosa beside #37

In September 2022, home-visit dental care resumed. At that time, the patient was unable to get out of bed and remained in bed throughout the day. Nutritional intake primarily occurred through the gastrostoma. The oral intake was limited to jellies and other light foods. During hospitalization, the patient did not wear dentures in either the maxilla or the mandible, as he had not worn them for a long time. Intra oral examination revealed ulceration of the buccal mucosa due to abrasion of the #44 locator abutment (Fig. 7). Therefore, we decided to remove the #34, #36, #37, #44, and #45 locator abutments and replace them with cover screws that were shorter and expected to prevent abrasion of the mucosa. Cover screws #34, #36, #44, and #45 were installed; however, implant fixture #37 was fractured at the implant collar region when the locator abutment was removed (Fig. 8). Because a cover screw could not be installed, the fractured implant was covered with composite resin in the implant fixture #37. However, none of these implants could sleep underneath the mucosa even after the cover screws were installed and the composite resin was filled. Unfortunately, new ulcers developed around sites #37 and #45 (Fig. 9). The patient continued to receive professional oral care and regular follow-up.

**Fig. 10:**
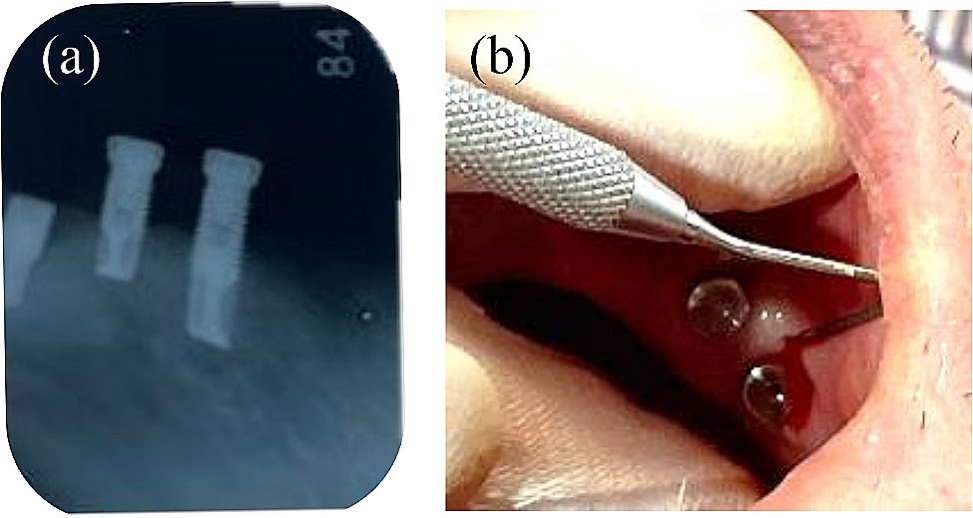
Recurrence of peri-implantitis at #45. (**a**) A dental radiograph of implants at #46 (left), #45 (center), and #44 (right). (**b**) Bleeding on probing the peri-implant sulcus at #45

In January 2022, the patient’s nursing care level further progressed to level 5. At that time, the #45 implant could not be observed because the right buccal mucosa completely covered the implant and the #45 cover screw. The patient experienced pain on touching the right buccal mucosa. Examination of the buccal mucosa revealed swelling of the peri-implant mucosa, drainage, and bleeding from the peri-implant sulcus. Dental radiographs indicated a diagnosis of recurrent peri-implantitis at #45 (Fig. 10). Professional care was initiated more frequently and included weekly brushing of the peri-implant sulcus with an internal brush, rinsing, and topical medication. After 1 month, drainage from the peri-implant sulcus was discontinued. However, in September 2022, the patient was readmitted to the hospital because of a urinary tract infection, and home-visit dental care was discontinued. The patient passed away 1 year after hospitalization.

## Discussion

We present a case in a patient with Parkinson’s disease, whose fixed implant prostheses were replaced with an IOD under nursing care. This is a valuable report because there are no previous reports on the progression of nursing care level after the replacement of FIP with an IOD.

When the patient underwent implant placement, he lived independently. During the 12 years between the ages of 65 and 77 years, his fixed implant-supported prostheses must have contributed to his quality of life as a professional vocalist.

In our patient, the onset of Parkinson’s forced a change in the dental treatment policy. This was because his general condition had deteriorated. His Hoehn and Yahl scale score was stage III, indicating mild to moderate disability with impaired postural reflexes. It was predicted that he would require long-term care in the near future as bradykinesia and rigidity progressed. Fixed prostheses cause less interference with singing but require greater therapeutic intervention when the remaining teeth are fractured due to parafunctions, such as the effects of crossbite or bruxism. Conversely, removable prostheses may affect singing ability; however, oral cleaning would be easier for caregivers, and functional restoration by denture restoration is considered easy, even when residual teeth are fractured horizontally. Therefore, in this case, after a thorough discussion with the patient and his wife, the decision was made to replace his fixed prostheses with a removable prosthesis, seeking ease of oral management as the level of nursing care progressed.

Replacing a fixed prosthesis with a removable prosthesis allows short-term functional recovery through denture repair in the event of a fracture of the remaining teeth during the nursing care period. Using a locator attachment allows for adjustment of the support and maintenance functions of the IOD according to the patient’s general condition. These advantages allow patients to maintain their oral intake, nutritional status, and singing ability until hospitalization. Owing to the deterioration of the patient’s general condition, the denture could no longer be worn, and the locator abutment was in continuous contact with the buccal mucosa, resulting in mucosal ulceration. The locator was replaced with a cover screw to reduce the height of the exposed implant site on the mucosa, and the interior of the fractured implant was filled with composite resin. However, the implants were not completely submucosal, and the mucosal injury did not heal. The failure to place implants submucosally may be attributed to low bone volume around the fixture or the use of an externally connected implant system. When selecting an implant system, systems that facilitate submucosal placement of implants should be considered, such as internally connected or bone-level implant systems. In patients who are expected to require long-term care in the future, it may be advisable to select only necessary implants during periods of outpatient visits and to consider prior removal of implants that cannot sleep submucosally.

The implant at #45 with recurrent peri-implantitis had already developed peri-implantitis during an outpatient visit. Visser et al. reported that a late-stage dementia patient with an IOD developed peri-implantitis because the superstructure was not removed for a long period, and a large amount of dental plaque and calculus remained around the implant site [12]. Additionally, the results of a questionnaire survey conducted by Kimura et al. indicated that more than 70% of caregivers lacked sufficient knowledge or skills regarding oral care for implants [13]. In this case, cleaning instructions were provided to his wife; however, cleaning the implant attachments, which were almost entirely covered by the buccal mucosa, was considered difficult for nonprofessional caregivers. Therefore, altering the oral environment to make it easier for non-specialist caregivers to clean is necessary. Thus, the replacement of a fixed implant prosthesis with an IOD requires not only the replacement of a fixed implant prosthesis with an IOD but also the removal of fixtures where peri-implantitis has occurred in the past.

## Conclusions

In conclusions, we attempted to address a case in which oral management had become difficult with the progression of the level of nursing care required, by replacing a FIP with an IOD in an older patient with Parkinson’s disease. Replacement with a removable prosthesis provided an environment that facilitated smooth oral care by caregivers and functional recovery in the event of trouble with residual teeth. However, it could not completely avoid the recurrence of buccal mucosal ulcers or peri-implantitis.

## Data Availability

No datasets were generated or analysed during the current study.

## Abbreviations

FIP: Fixed Implant-supported Prostheses
IOD: Implant Overdenture
